# Porcelain Impression Cups for Taking Impressions of the Teeth

**Published:** 1873-08

**Authors:** B. M. Wilkerson


### ARTICLE III.

### *Porcelain Impression Cups for Taking Impressions of the Teeth.*

BY B. M. WILKERSON, D. D. S., M. D.

Porcelain impression cups are little used, notwithstanding they are in many cases superior to the metal. They are much more attractive to the eye of the patient, as the practitioner has no trouble to keep them beautifully clean. They are excellent for taking partial, as well as full impressions; and especially where there are undercuts, as the cup will leave the plaster (after it has set,) with little force of the hand; then the operator can easily break away the impression, replace it in the cup, and run his model. When it is desirable to build up the arch with wax, and when there are undercuts requiring the removal of the cup before the impression, it is best not to stick it tightly, nor cover too much of the palatine surface of the cup, as it will be difficult to remove from the impression. The cup can be made as deep as desired by extending the rim with sheet wax.

The cups *must* be made *perfectly smooth*, with *no undercuts.* They are of sufficient strength for all practical purposes. A thorough trial by a neat operator will insure their use a permanency in his practice, and give general satisfaction to his patients.

